# Amplification and up-regulation of MIR30D was associated with disease progression of cervical squamous cell carcinomas

**DOI:** 10.1186/s12885-017-3201-0

**Published:** 2017-03-29

**Authors:** You Zhou, Yinghua Hao, Yuxia Li, Ruizhen Li, Ruifang Wu, Shubin Wang, Zhengyu Fang

**Affiliations:** 1 Biomedical Research Institute, Shenzhen Peking University- The Hong Kong University of Science and Technology Medical Center, Shenzhen, Guangdong Province 518036 China; 2 Department of Gynecology and Obstetrics, Peking University Shenzhen Hospital, Shenzhen, Guangdong Province China; 3 Department of Medical Oncology, Peking University Shenzhen Hospital, Shenzhen, Guangdong Province China

**Keywords:** Cervical squamous cell carcinoma, miR-30d, MIR30D, Copy number variation, Gene expression

## Abstract

**Background:**

Cervical squamous cell carcinoma (CSCC) is the most frequent type among cervical cancers. Although the altered miRNA miR-30d expression and the amplified chromosome locus of MIR30D, 8q24, have been reported in somatic cancers, the definitive functional impact of such region especially in CSCC remains under-investigated.

**Methods:**

One hundred thirty-six cases of CSCC tissues and matched adjacent normal ovarian epithelial tissues were assessed in this study. FISH and qPCR were performed to detect the copy number and microRNA expression of MIR30D gene in the collected samples. In in-vitro study, proliferation of CSCC cells were analyzed using WST-1 assay and invasion abilities of CSCC cells were evaluated by transwell assay. In-vivo study using a model of nude mice bearing tumor was also performed.

**Results:**

Copy number gains of MIR30D were detected in 22.8% (31 out of 136) of CSCC samples. Copy number of MIR30D was positively correlated with tumor progression. CSCCs with lymph node metastases (LNM) also showed more frequencies (36.4%) of MIR30D amplification than those without LNM (18.4%, *p* < 0.05). CSCCs with increased copy number of MIR30D also showed a positive correlation with miR-30d up-regulation. Inhibition of miR-30d in CSCC cells led to impaired tumor growth and migration.

**Conclusions:**

Copy number amplifications of MIR30D gene and enhanced expression of miR-30d were positively correlated with tumor progression in CSCCs, indicating miR-30d might play an oncomiric role in the progression of CSCC.

**Electronic supplementary material:**

The online version of this article (doi:10.1186/s12885-017-3201-0) contains supplementary material, which is available to authorized users.

## Background

Invasive cervical cancer is one of the leading causes of cancer-related death in gynecological tumors [1–4]. The exploration of new strategies for diagnoses, treatment, and prognoses of cervical squamous cell carcinomas (CSCCs) merit special attention [5]. About 80% to 90% of cervical cancers are squamous cell carcinomas [6, 7], where the abnormal squamous cells develop and cover the surface of the cervix. Although 80%–95% of women with early-stage CSCC could benefit from traditional surgery and chemoradiotherapy, it remains hard to reduce the recurrence- and metastasis-related cancer death [8–10].

MicroRNAs (miRNAs) are a class of short non-coding RNAs that negatively regulate the expression of their protein-coding mRNA targets [11, 12]. Up to now, thousands of miRNAs in human have been discovered. Despite their relatively limited number, each individual miRNA can alter the expression of hundreds of targeted mRNAs [13]. Therefore, miRNAs are considered as major regulators of many important biological processes including apoptosis, viral infection and cancer development [14–17]. Whole genome analyses showed that approximately half of miRNA coding genes lie in fragile sites or in tumor-associated genomic regions [18]. Recently, dys-regulation of microRNA expression has been found to be one of the abnormal events during the development of cervical cancer [19–21].

miR-30d is fairly frequently overexpressed in many human epithelial cancers and functionally affects various tumor biological events such as proliferation, differentiation, metastasis, apoptosis, etc. [22–25]. Consistently, the chromosome locus of MIR30D gene, 8q24, is also found frequently amplified by comparative genomic hybridization (CGH) detection in various types of somatic cancers [26, 27].

Although overexpression of miR-30d in cervical cancers was reported in a previous study using a high throughput assay [28], the case number was very limited (*n* = 10). Importantly, the clinical significance of miR-30d in the progression of cervical cancers remains under-investigated. In this research, 136 sporadic CSCC tumor samples and their matched adjacent normal tissues (ANTs) were collected from a Chinese population. Copy number variations (CNVs) of MIR30D gene as well as expression levels of miR-30d were examined, and analyzed with clinical characterization. In-vitro studies were also performed to estimate the role of miR-30d in the cell proliferation and migration of CSCCs. Our findings showed that amplified copy number of the MIR30D gene and/or up-regulated expression of miR-30d were positively correlated with CSCC disease progression, indicating that miR-30d plays as a critical oncomir in CSCC progression and could be a potential biomarker and therapeutic target for CSCCs.

## Methods

### Patients and tissue collection

Samples were taken from CSCC patients at the Department of gynecology and obstetrics, Peking University Shenzhen Hospital from June 2008 to July 2014. A summary of cohort characteristics was shown in Table 1. Tumors were staged according to the classification system: Stage 0 (The carcinoma is confined to the surface layer of the cervix; not included because it cannot be distinguished from CIN3), Stage 1 (The carcinoma has grown deeper into the cervix, but has not spread beyond it, *n* = 35), Stage 2 (Cervical carcinoma invades beyond the uterus, but not to the pelvic wall or to the lower third of the vagina, *n* = 78, IIa = 47, IIb = 31), Stage 3 (The tumor extends to the pelvic wall and/or involves lower third of the vagina and/or causes hydro-nephrosis or non-functioning kidney, *n* = 16) and Stage 4 (The carcinoma has extended beyond the true pelvis or has involved (biopsy proven) the mucosa of the bladder or rectum, *n* = 7). Cases under Stage IIa (including IIa) were grouped into early-stage, whose samples were collected from surgery. Cases above IIb (including IIb) were grouped into advanced stage and received radiotherapy and chemotherapy, whose samples were collected from biopsy. The matched ANTs were defined as tissues located at least 1.5 cm far from the macroscopically unaffected margins of the tumor. The samples without qualified ANTs were excluded. All samples were rapidly frozen in liquid nitrogen immediately after excision and were stored in liquid nitrogen until use.

**Table 1:** Summary of the cohort characteristics

Characteristics	Information	
Age	Average age	49.8
	Range	18 ~ 71
Risk factors	Multiple sex partners	55
	Multiparous or pregnant at youth	26
	First sex before 18	19
	None of above	36
FIGO Staging	I	35
	II	78
	III	16
	IV	7
HPV infection	High risk (HPV16, 18 or both)	99
	Other HPVs	37
Lymph node metastasis(LNM)	With LNM	33
	Without LNM	103
Smoking history	Yes	21
	No	115

### RNA extraction and quantitative real-time PCR (qPCR)

Total RNA from tissues or cell lines was isolated in accordance with the manufacturer’s instruction of the AxyPrep™ Blood Total RNA MiniPrep Kit (Axygen). Then the first-strand cDNA was synthesized by reverse transcription with the RevertAid™ First Strand cDNA Synthesis Kit (Fermentas). And the primer for reverse transcription of miR-30d is: 5′-GTC GTA TCC AGT GCA GGG TCC GAG GTA TTC GCA CTG GAT ACG ACC TTC CA-3′.

Bio-Rad iQ™ SYBR Green Supermix real-time PCR kit and CFX96 Detection System was used to perform qPCR. The quantitative primer pair for miR-30d is: forward: 5′-GCA TTG TAA ACA TCC CCG AC-3′, and reverse: 5′- GTG CAG GGT CCG AGG TAT TC-3′. Melt curves were produced for product identification and purity at the end point of PCR cycles. Because of the near-100% amplification efficiencies of all targeted genes, qPCR results were calculated using 2^−ΔΔct^ method. miR-30d expression level in each sample was analyzed using Bio-Rad CFX Manager software and normalized to the expression of U6. The expression levels of the target miRNAs or mRNAs were visually identified using exploratory data analysis with scatter plots [29]. Each quantitative reaction was replicated twice, and the average value was used in the scatter plot. Other primer pairs for the targeted mRNA are listed in Table 2.

**Table 2:** Primer sets for the targeted mRNAs

Gene	Forward	Reverse
ATG12	TTGTGGCCTCAGAACAGTTG	GAGAGTTCCAACTTCTTGGTCTG
GNPDA1	TGCCTGTTTGAAGCTACTGC	ACCAAACATAGCCCTTAGGC
CASP3	AGGACTCTAGACGGCATCCA	TGACAGCCAGTGAGACTTGG
CCNE2	TAATAAGGCTTAGATGAACATGGTG	AGTTAGGAAGGAGCCACAGC
GALNT1	TTGTGCCTAAGAATGTTTCCA	CCCATGTGCTTGATGTTGAT
GNAI2	GACCATCTGCTTCCCTGAGT	TGGTGTCTTTGCGCTTATTC
FOXO3	TGATTTGAAGCACCTCATCC	TTAAGAAAGGCGGCAGAGTT
SNAI1	TCTGGTTCTGTGTCCTCTGC	GACAGGCCAGCTCAGGAAT
SOCS1	CGACTACCTGAGCTCCTTCC	AACACGGCATCCCAGTTAAT
SPRR2D	CTGTAGTACACATCACTTGTGGC	ACTTGCATCCCAGGACAGAT
GAPDH	TCCAAAATCAAGTGGGGCGA	TGATGACCCTTTTGGCTCCC
ACTB	GACCTGACTGACTACCTCATGAAGAT	GTCACACTTCATGATGGAGTTGAAGG

### DNA extraction and quantification of copy numbers

The method of copy number calculation has been introduced previously [30, 31]. Genomic DNA was prepared from tissues following the protocol of the Genomic DNA Extraction Kit (Innocent, Shenzhen, China). Quantitative PCR was performed through Bio-Rad CFX96 Detection System. The relative copy numbers of MIR30D were normalized to RNAse P gene (copy numbers =2) and analyzed by the comparative Ct method. The copy numbers as 0, 1, 2 and 3 were respectively defined by cut-off values of 0.25, 0.75, 1.25 and 1.75. The primer pair, forward: 5′-GAT GAT GAC TGG CAA CAT-3′ and reverse: 5′-GAA TAG CCG GTA GCA GCA-3′, was used for the detection of MIR30D. And the primer pair for RNAse P is: forward: 5′-AGA CTA GGG TCA GAA GCA A-3′ and reverse: 5′-CAT TTC ACT GAA TCC GTT C-3′. The relative copy number fold-change was calculated using the 2^−ΔΔCt^ method.

### Fluorescence in situ hybridization (FISH) analysis

CSCC tissues and matched ANTs were collected in pairs as stated before, and ten pairs (5 with MIR30D amplification, 5 with unaltered MIR30D copy number) were selected for FISH analysis. The tissue was minced into single-cell suspension with a scalpel after treatment with 0.075 M KCl for 10 min. Then the cell suspension was fixed in a fixative (3:1 ratio of methanol and acetic acid). Target slides were prepared by dropping the suspension of isolated fixed nuclei on a glass slide, and slides were fixed in 70 °C steam and denatured in 2× SSC/70% formamide, pH 7, at 75 °C for 5 min and dehydrated in graded ethanol.

FISH detections were performed with dual-labeling hybridization using a directly labeled centromere probe for chromosome 8 (Spectrum Green-labeled) together with a probe for the MIR30D locus (8q24.22; Spectrum Orange-labeled). After denatured at 75 °C for 5 min, probes were hybridized onto the target slides overnight at 37 °C. Then these slides were washed with 50% formamide/2× SSC for 10 min three times, 2× SSC for 5 min, and 2× SSC/NP40 for 5 min at 45 °C. After washing, the slides were counterstained with 1 μg/ml DAPI, and signals for each locus-specific FISH probe were assessed using an Olympus microscope equipped with a triple band pass filter. At least 300 nuclei were examined in each sample. FISH signals were counted and recorded as 0, 1, 2, 3, 4, 5, or more signals for each probe.

### Cell culture and proliferation assay

Human cervical cancer cell lines HeLa, C4–1, SiHa, Caski and C-33A were obtained from the Cobioer Biosciences Co., Ltd. (Shanghai, China) and grown in Dulbecco’s modified Eagle’s medium (DMEM, Gibco) supplemented with 10% FBS (PAA) and 1% penicillin/streptomycin (Life Technologies, Inc.) at 37 °C and 5% CO_2_.

WST-1 measurement was used to detect cell proliferation. Cells transfected with mimic, inhibitor or non-sense strand were seeded onto 96-well culture plates with 1 × 10^3^ cells per well, The proliferation of cells was tested using the colorimetric reagent WST-1 (Roche) at different time points (0, 1, 2, 3, 4, and 5 days).

### Cell invasion assay

Invasion assays were done using 24-well transwell chambers (8 μm pore size; BD Biosciences). After incubation with 1% BSA for 1 h at 37 °C, transwells were coated with fibronectin (10 mg/ml in PBS) overnight at 4°C. Meanwhile, SiHa and HeLa cells were transfected with miR-30d mimic, inhibitor or control strand for 24 h. Then the cells were collected by trypsin-EDTA digestion, washed once in 10% FBS/DMEM, and resuspended in 1% FBS/DMEM at 2 × 10^5^ cells/ml. And the cell suspensions were equivalently added to the upper compartment of each chamber (100 μl per chamber). Following 12 h incubation, the non-invaded cells on the upper surface of the membrane were removed with a cotton swab, whereas the cells that had invaded through the membrane to the underside surface were fixed by 3% formaldehyde and stained with 0.3% crystal violet for 10 min. The cells on the underside of the membrane were counted and the number of cells in five different fields (×100 magnification) was used to quantify cell invasion. Data represent the average ± SD of three independent experiments.

### Retroviral transduction

Each retroviral vector and the pLC 10A1 retroviral packaging vector (Imgenex, San Diego, CA, USA) was co-transfected into HEK293T cells using the Lipofectamine LTX reagent (Invitrogen). After 24 h, the conditioned medium was collected as a viral solution. The retroviral vectors were infected into the cells in the medium that contained 10 μg/mL polybrene (Sigma, St. Louis, MO, USA) and allowed to incubate for 24 h. Then, the viable cells were selected using 800 μg/mL neomycin (G418; Invitrogen). The selected pooled clones were used in the biological analyses. The transfection efficiency was determined using qPCR analysis.

### Generation of the in vivo xenograft model

Five-week-old male nude mice were used in this study. Subconfluent HeLa and SiHa cells were transduced with miR-30d-blocking or control viral vectors, trypsinized, and suspended in Phosphate-buffered saline (PBS). Then, the cells were subcutaneously injected into the right (to inhibit miR-30d) and left (control) flanks of the same mice. HeLa was subcutaneously injected at a concentration of 1 × 10^6^ cells. SiHa cells were subcutaneously injected as a mixture of 2 × 10^6^ cells and an equal volume of Matrigel (BD Biosciences), reaching a total concentration of 10 mg/mL (15 mice each group). Tumor growth was followed for 42 days after tumor cell injection. Moribund animals were euthanized according to the protocols of the Peking University Health Science Center. Each xenograft tumor volume was calculated using the following formula: tumor volume = (short axis^2^ × long axis)/2.

### Gene expression studies

Gene expression profiles were obtained by using Affymetrix GeneChip^®^ Probe Arrays. According to the manufacturer’s instructions, the total RNA was isolated from each sample (prewashed by 50 mM potassium phosphate buffer, pH 7.4) with RNeasy Mini Kit (Qiagen). And Bioanalyzer 2100 (Agilent Technologies, US) was applied to confirm the quality of extracted RNA. The DNA microarray data were was produced by Bio Matrix Research (Chiba, Japan).

GeneSpring software package (Agilent Technologies, US) was used for statistical analysis, and online tools of the Munich Information Center for Protein Sequence (MIPS) was used for the pathway- or function-based classification. Gene expression data have been submitted to the Gene Expression Omnibus (GEO; http://www.ncbi.nlm.nih.gov/geo).

### Statistical analysis

The categorical data were analyzed for statistical significance by chi-square test or Fisher exact test, or analysis of variance (ANOVA). All the above mentioned analyses were performed using GraphPad Prism 5.0 statistical software and *p*-values less than 0.05 were considered statistically significant.

## Results

### Enhanced expression of miR-30d in CSCCs was correlated with tumor progression

**Fig. 1:**
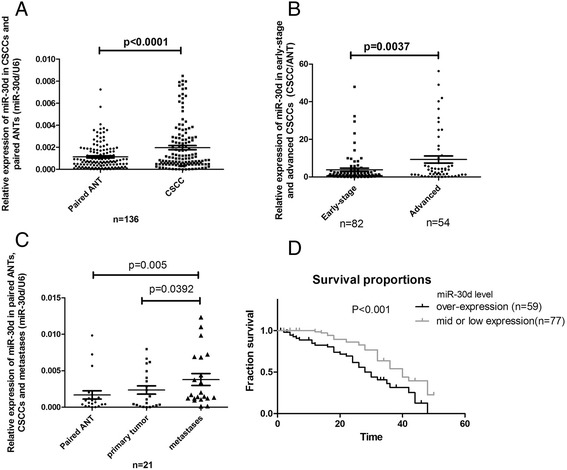
Relative expression of miR-30d in CSCCs. qPCR assay was carried out as described under Materials and Methods section, and the results were obtained from the indicated group of samples. **a** Scatterplot illustrated the relative expression level of miR-30d as a ratio of miR-30d to U6 in all the CSCC samples compared with ANTs. **b** Scatterplot illustrated the relative expression of miR-30d as a ratio of CSCC to paired ANT in the CSCCs at different stages. **c** Scatterplot illustrated the relative expression of miR-30d as a ratio of CSCC to paired ANT in the primary tumors and lymph node metastases. **d** Kaplan-Meier and log-rank analysis (*n* = 136). The 2 groups were divided according to the expression levels of miR-30d (over-expression group, T/*N* ≥ 2, *n* = 57; mid or low expression group, *n* = 79) and analyzed (*P* = 0.0009; log-rank test) to determine its association with biochemical recurrence in CSCC

The transcriptional expression of miR-30d was evaluated using qPCR. Compared to ANTs, the expression levels of miR-30d were markedly enhanced in the collected CSCC samples (*p* < 0.001), as shown in Fig. 1a. In addition, there were statistically higher expression level of miR-30d in the group of advanced CSCCs (*n* = 54) than early-stage CSCCs (*n* = 82, *p* = 0.0037, Fig. 1b).

To further investigate the role of miR-30d abnormalities in the spread of CSCCs, we compared the expression of miR-30d between primary tumors and metastases isolated from 21 cases with qualified paired lymph node metastasis samples (≦IIa, from surgery). As shown in Fig. 1c, paired t-test analyses showed that metastatic CSCCs had a slight but statistically significant increase of miR-30d expression, in comparison with primary tumors (*p* = 0.0392).

To explore the clinical significance of altered miR-30d expression levels, the intracellular miR-30d expression were scored as overexpression (CSCC/ANT ≧2, *n* = 59) and moderate or low expression (*n* = 77). It showed that, increased expression level of mature miR-30d had very significant correlation with poor clinical outcomes in CSCC patients (Fig. 1d, P =0.0013).

### Gene copy number gains of MIR30D in CSCC samples

As shown in Table 3, distribution of MIR30D copy number in ANTs had no statistical difference in comparison to healthy normal controls (HNCs), and thus the ANTs could be used by the present study as matched controls for the CSCC tissues.

**Table 3:** Comparison of CNVs^a^ of MIR30D between adjacent normal tissues and healthy normal controls

Samples	n	Copy number					*P* (vs. HNC)
		Deletion			Amplification		
		0	1	2	3	>3	
HNC^a^	168	1	7	155	3	2	---
ANT^a^	136	2	1	127	4	2	0.747

^a^ *CNV* copy number variation, *ANT* adjacent normal tissue, *HNC* healthy normal control

In a total of 136 matched samples of CSCC patients, we had examined the CNVs of MIR30D in cancer tissues and ANTs (Table 4). Copy number gains of MIR30D gene were found in a portion of CSCC tissues (22.8%, 31 out of 136). Much higher frequencies of MIR30D gene amplification were observed in the advanced CSCCs (31.6% for stage3–4) than those in early-stage CSCCs (16.5% for stage 0–2). These results indicated that copy number gains of MIR30D gene were positively correlated with CSCCs tumor progression (*p* < 0.01).

**Table 4:** CNVs^a^ of MIR30D gene in CSCC tissues and matched ANTs^a^

Population		Number	Copy numbers		P (vs. ANT)	P (vs. early-stage CSCC)
			Deletion or Unalteration	Amplification		
			≦2	>2		
Total	ANT	136	130	6	---	---
	CSCC		105	31	9.79E-06	---
Early-stage (0-IIa)	ANT	82	80	2	---	---
	CSCC		68	14	2.83E-3	---
Stage 3–4 (IIb-IV)	ANT	54	50	4	---	---
	CSCC		38	17	8.92 E-4	0.038

^a^ *CNV* copy number variation, *CSCC* cervical squamous cell carcinoma, *ANT* adjacent normal tissue

In the collected CSCC cases, 33 out of 136 showed LNM. We next analyzed the copy number of MIR30D in the CSCCs with or without LNM. As shown in Table 5, 36.4% of CSCC cases with LNM showed MIR30D amplification, which was much higher than those without LNM (18.4%, *p* = 0.0327). However, in the 21 CSCC cases with qualified lymph node samples, only 2 cases showed increased MIR30D copy number in LNMs compared to the primary tumors while others had no differences between the LNMs and primary sites. Nevertheless, MIR30D amplifications might play a role in CSCC metastasis.

**Table 5:** CNVs^a^ of MIR30D gene in CSCCs with or without LNM

Population		Number	Copy numbers		P (vs. ANT)	P (vs. early-stage CSCC)
			Deletion or Unalteration	Amplification		
			≦2	>2		
Total	ANT	136	130	6	---	---
	CSCC		105	31	9.79E-06	---
Without LNM	ANT	103	99	4	---	---
	CSCC		84	19	1.95E-3	---
With LNM	ANT	33	31	2	---	---
	CSCC		21	12	6.73E-3	0.0327

^a^ *CNV* copy number variation, *LNM* lymph node metastasis, *CSCC* cervical squamous cell carcinoma, *ANT* adjacent normal tissue

**Fig. 2:**
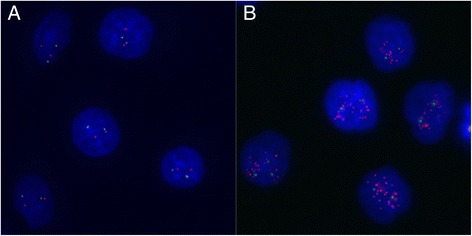
Gene amplification of MIR30D in CSCCs. Representative figures of FISH analysis using chromosome 8q specific alpha satellite DNA probe and chromosome 8q24 specific probe for MIR30D. **a** Nucleus of ANT tissue with two signals for each of green and red, showing no amplification of chromosome 8q or MIR30D gene; **b** Nucleus of CSCC tissue with normal signals for green and multiple signals for red, indicating relative amplification in chromosome 8q24 or MIR30D gene

In order to verify the CNVs of MIR30D gene in CSCCs, paraffin-embedded CSCC tissues and matched ANT tissues were picked out (*n* = 10, 5 amplification, 5 unaltered) to conduct a reexamination by FISH analysis with chromosome 8q and 8q24 specific probe (MIR30D). The obtained results showed highly consistence with those from the qPCR experiments (Fig. 2 and Table 6).

**Table 6:** FISH results in 10 CSCC/ANT pairs

Results from real-time PCR analysis	Case number	Stage	CSCC		ANT		MIR30D gene amplification Ratio (T/N)
			Gains on 8q24 (MIR30D)	Gains on 8q	Gains on 8q24 (MIR30D)	Gains on 8q	
Unaltered MIR30D copy number	1	2	−	−	−	−	0.97
	2	3	−	−	−	−	1.03
	3	3	−	−	−	−	0.92
	4	3	−	−	−	−	1.15
	5	4	−	−	−	−	1.02
MIR30D amplification	6	2	+	−	−	−	16.75
	7	3	+	−	−	+	8.48
	8	4	+	+	−	−	3.62
	9	4	+	−	−	−	22.43
	10	4	+	+	−	−	9.67

### Positive correlation between amplifications of MIR30D gene and miR-30d up-regulation in CSCCs

**Fig. 3:**
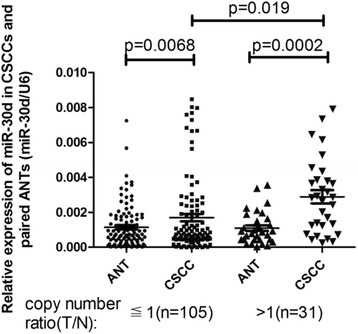
MIR30D amplification leads to overexpression of miR-30d. Scatterplot illustrated the relative expression level of miR-30d as a ratio of miR-30d to U6 in the groups with or without MIR30D amplification

Gene CNVs are frequently associated with the quantitative as well as functional shift of their gene products. We then tested whether the expression levels of miR-30d were correlated with gene copy alterations in several selected samples with amplified or unaltered copies of MIR30D gene. As in Fig. 3, in both groups with amplified or unaltered copies of MIR30D, the CSCC tissues showed significantly higher expression of miR-30d than ANTs (*p* < 0.005). It’s interesting that the CSCC samples of MIR30D amplified group showed a statistical difference of miR-30d expression compared to unaltered group (*p* = 0.019). Hence, to some extent DNA copy amplifications were the driving force of the up-regulation of miR-30d in CSCCs.

### miR-30d plays an oncomiric role in CSCC cells

**Fig. 4:**
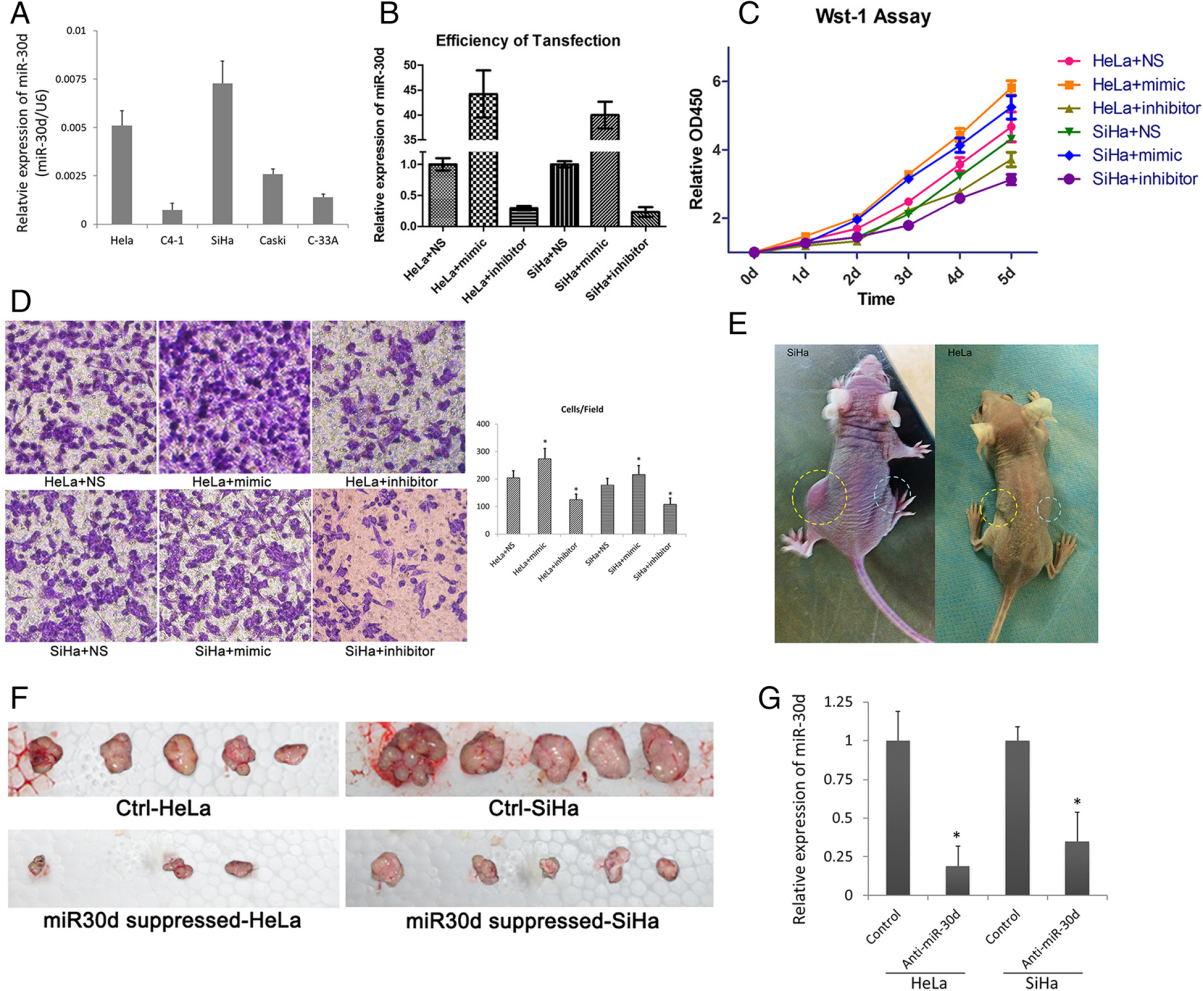
miR-30d acts as an oncomir in CSCCs. **a** Expression levels of miR-30d were examined by real-time PCR; **b** After treatment of miR-30d inhibitor, mimic and control strand, the expression levels of miR-30d were examined by real-time PCR. The relative expression of miR-30d is illustrated as a ratio to control (U6); **c** WST-1 (Roche) assay measuring the activity of mitochondrial dehydrogenases was performed following the manufacturer’s instruction at 0-, 1-, 2-, 3-, 4- day time points. The results were obtained from three independent experiments. Error bars represent the standard deviation of the mean; **d** Cell migration was determined using a transwell assay as described in the Materials and methods section. Microscopic image of migrated HeLa and SiHa cells with indicated treatments. Diagrams of migrating cells from the different transfectants are shown, which are from more than three independent experiments.*p < 0.05 versus control. **e**-**f** Tumor growth indicates the stable inhibition of miR-30d expression in CSCC cell lines when subcutaneously injected into the right (inhibitor of miR-30d) and left (control) flanks of male nude mice (*n* = 15). Tumor growth was followed for 42 after tumor cell injection. The dotted line represents the tumor mass. **g** Expression of miR-30d extracted from xenografts using qPCR. The expression level of miR-30d was normalized to the expression state of U6. Values represent the means, and the error bars represent the SD. *p < 0.05 according to the paired t-test

Although miR-30d was considered as an oncomir in various kinds of epithelial cancer [24, 32, 33], it has also been reported that miR-30d suppresses cell proliferation and motility and induces apoptosis in several types of tumors [34, 35]. In order to determine the role of miR-30d in CSCC, multiple corresponding cell lines including HeLa, C4–1, SiHa, Caski and C-33A were first evaluated for miR-30d expression. HeLa and SiHa showed relatively higher expression of miR-30d (Fig. 4a), and thus were selected for the following knockdown experiments.

After transfected with the mimic or inhibitor of miR-30d, the expressions of miR-30d in the above cell lines were significantly altered (Fig. 4b). The effect of altered expression of miR-30d on CSCC cell proliferation was estimated by WST-1 assay. As shown in Fig. 4c, the expression of miR-30d was positively correlated with proliferation rates of the CSCC cells.

Next, the migration abilities of HeLa and SiHa cells transfected with miR30d mimic, inhibitor or non-sense strand were estimated by the trans-well assay. A positive correlation between miR-30d expression and CSCC cell migration was also observed (Fig. 4d). Thus, enhanced expression of miR-30d might play a role in the progression of CSCCs.

To evaluate the functional effects of miR-30d in vivo, we established 2 CSCC cell lines (HeLa and SiHa) that stably suppressed miR-30d expression via retroviral transduction. These miR-30d-suppressed cell lines were subcutaneously injected into the right side of male nude mice; control CSCC cell lines were simultaneously injected into the left side (15 mice each group). The representative xenograft mice and xenografted tumors were shown in Fig. 4e and f, respectively. At sacrifice, the mean volumes of tumor xenografts from the nude mice were measured (Table 7), which showed that the CSCC cells with suppressed expression of miR-30d formed significantly smaller tumor nodules compared with the controls. The attenuation of miR-30d expression in these xenografts were also confirmed by qPCR (Fig. 4g). Taken together, these results suggest that up-regulation of miR-30d may promotes CSCC cell proliferation and migration contributing to tumor progression and metastasis.

**Table 7:** Mean final volume of xenografts when nude mice sacrificed

	SiHa (mm^3^)	HeLa (mm^3^)
Control (*n* = 15)	892.037 ± 329.843	496.234 ± 219.317
miR-30d suppressed (*n* = 15)	342.902 ± 152.035	204.298 ± 98.953

### miR-30d regulates the expression of a number of genes in CSCCs

Since a single miRNA can regulate tens or hundreds of targeted mRNA, so even though the expression status of only one single miRNA was altered, cells may undergo a great phenotypic change. Oncomiric role of miR-30d would be performed by transforming multiple signaling pathways rather than by disturbing one or a few cancer-associated genes. After the transfection of a miR-30d mimic into HeLa and SiHa cells, microarray analyses were used to display the transcriptional changes. Over-expression of miR-30d down-regulated 464 and 376 genes in HeLa and SiHa cells, respectively [see Additional files 1 & 2].

**Fig. 5:**
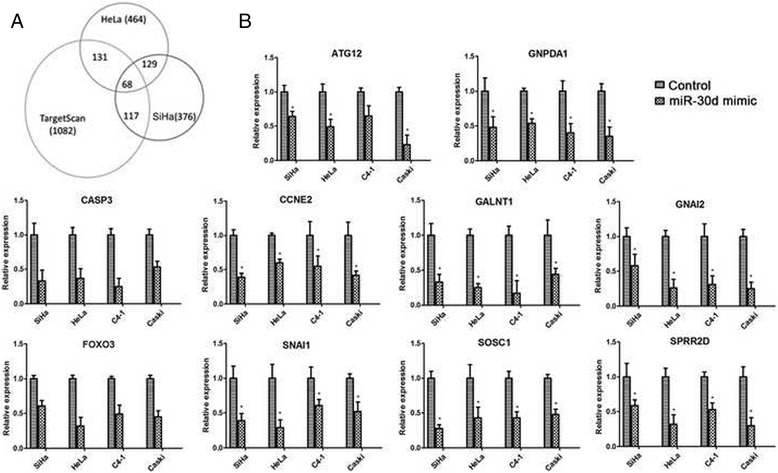
miR-30d regulates various kinds of cancer-related genes. **a** Venn diagrams of transcript numbers shared by downregulated transcripts in miR-30d mimic transfections in HeLa and SiHa cells and predicted targets of miR-30d by TargetScan. **b** The microarray results were validated by real-time RT-PCR in 4 tumor cells. qPCR validation of transcripts that were downregulated in both HeLa and SiHa cells after transfection with the miR-30d mimic and that were also predicted miR-30d targets by TargetScan. Validations were done in SiHa and HeLa cells, as well as in 2 independent cell lines, C4–1 and Caski., *p < 0.05

Bioinformatics methods were used to analyze the possible direct targets of miR-30d. By TargetScan (http://www.targetscan.org/), 131 of 464 down-regulated genes in HeLa and 117 of 376 in SiHa were predicted as targets of miR-30d (Fig. 5a). These data suggested that about 30% of those downregulated transcripts were indeed directly repressed by miR-30d. Moreover, about 1/3 (*n* = 129) down-regulated genes were shared in both cell lines, and 68 of these were TargetScan predicted targets of miR-30d [see Additional file 3]. Notably, several known miR-30d targets identified by other groups, including ATG12 [25], CASP3 [32], SNAI1 [36], SOCS1 [24], FOXO3 [34] and GNAI2 [37], were consistently found in our 68 transcript list. Finally, 10 of these genes were selected and further verified by qPCR in HeLa and SiHa cells, as well as another 2 independent cell lines, C4–1 and Caski (Fig. 5b). Taken together, these results indicate that miR-30d directly affects the expression of a number of genes in CSCC to play its oncomiric role.

## Discussion

Besides identification and functional annotation of miRNAs, investigation of transcriptional regulation of miRNA genes should also be one of the notable issues. In the present study, we found that copy number variation also played a role in the dysregulated expression of cancer-microRNA in CSCCs. The amplifications of MIR30D (22.8%, 31 out of 136) were found in collected CSCC samples. Given that no statistical difference of MIR30D CNVs between HNCs and ANTs was observed, the CNVs of MIR30D were more likely to acquire aberrations in CSCC tumor tissues. However, the frequency of MIR30D gene copy number gain was lower than previously reported proportions of chromosomes 8q24 gain as 36–57% in somatic cancers [7, 38, 39]. This discrepancy might be due to the limited region of MIR30D gene on chromosome 8q24 which is less influenced by repeated replication during tumor progression. Amplifications of MIR30D were mainly found in advanced CSCCs, indicating that the increase of MIR30D copies might occur in the progression but not the initiation of CSCCs and may contribute to tumor aggressiveness. We also found that CSCCs with LNM showed more frequencies of MIR30D amplification than those without LNM, which indicated the potential association between MIR30D amplification and CSCC metastasis. Interestingly, in 2 cases with LNM, we found that the copy numbers of MIR30D were increased in LNMs compared to primary tumor. Did the copy number gain of MIR30D take place in migrating cells at the initiation of metastasis or later in the lymph node in these 2 cases? This needs further investigation.

Microarray-based comparative genomic hybridization (aCGH) analyses have shown that CNVs may directly or indirectly make a healthy body susceptible to cancer by altering the expression of oncogenes or tumor suppressor genes. Detection of the CNVs status is just a starting point for investigations into the role of such gene alterations in the development of cervical cancer. However, there are many discrepancies among the results obtained from the previous high-throughput studies of CNVs. Therefore, it is necessary to validate these CNVs in a large number of clinical samples. Furthermore, the detection sensitivity could also be improved by using sequence specific quantitative PCR to examine short DNA fragments of hundreds of base pairs.

Although it has been thought that cell phenotype is well correlated with the genotype of CNVs [40, 41], our study on the correlation between expression of miR-30d and copy numbers of MIR30D gene showed discordant findings. Compared to ANTs, the expression of miR-30d in CSCC tissues was increased in both groups with or without MIR30D amplification. Thus CNVs were not the only motivating factor for over-expression of the miR-30d in CSCCs. Some other mechanisms could be involved in the transcriptional regulation of miRNA expression. For instance, CpG island hyper-methylation have been reported to be involved in the regulation of miR-30d expression in somatic malignancies.

In the in-vitro and in-vivo studies, we showed that amplification and up-regulation of miR-30d promoted CSCC growth and metastasis, which further indicated the important role of miR-30d de-regulation in the progression of CSCC. As a non-coding RNA, miR-30d must mediate its tumor-promoting role through suppression of special targets. Here we also screened several key targets of miR-30d that might be involved in this progress. In the genes suppressed by miR-30d over-expression, most are tumor-suppressing genes that were down-regulated by miR-30d transfection. However, several target genes, such as CCNE2 and SNAI1, are positively correlated with tumorigenesis. These indicated the complex role of microRNAs in influencing tumorigenesis.

## Conclusions

To summarize, our results demonstrate that the amplification of MIR30D copy number were present in a certain proportion of CSCC cases and were positively correlated with its transcriptional expression as well as progression of tumor. Enhanced expression of miR-30d plays an oncomiric role in CSCC through the regulation of various cancer-related genes.

## Additional files

Additional file 1: Down-regulated genes in HeLa cells by mir-30d mimic transfection. Over-expression of miR-30d down-regulated 464 genes in HeLa cells. (XLS 76 kb)

Additional file 2: Down-regulated genes in HeLa cells by mir-30d mimic transfection. Over-expression of miR-30d down-regulated 376 genes in SiHa cells. (XLS 64 kb)

Additional file 3: List of the shared downregulated genes by miR-30d mimic transfection in Hela and SiHa cell lines, as well as the TargetScan-predicted miR-30d targets. One hundred twenty-nine down-regulated genes were shared in Hela and SiHa cell lines, and 68 of these were TargetScan predicted targets of miR-30d. (XLS 30 kb)

## Abbreviations

ANT: Adjacent normal tissue
CNV: Copy number variation
CSCC: Cervical squamous cell carcinoma
DMEM: Dulbecco’s modified Eagle’s medium
FISH: Fluorescence in situ hybridization
HNC: Healthy normal control
LNM: Lymph node metastasis
miRNA: microRNA
